# Sequence variants of the *DFNB31* gene among Usher syndrome patients of diverse origin

**Published:** 2010-03-23

**Authors:** Elena Aller, Teresa Jaijo, Erwin van Wijk, Inga Ebermann, Ferry Kersten, Gema García-García, Krysta Voesenek, María José Aparisi, Lies Hoefsloot, Cor Cremers, Manuel Díaz-Llopis, Ronald Pennings, Hanno J. Bolz, Hannie Kremer, José M. Millán

**Affiliations:** 1 Unidad de Genética, Hospital Universitario La Fe. Valencia, Spain; 2 CIBER de Enfermedades Raras (CIBERER), Valencia, Spain; 3 Dept Otorhinolaryngology, Head and Neck Surgery, Radboud University Nijmegen Medical Centre, Nijmegen, The Netherlands; 4 Dept Human Genetics, Radboud University Nijmegen Medical Centre, Nijmegen, The Netherlands; 5 Nijmegen Centre for Molecular Life Sciences, Radboud University Nijmegen, Nijmegen, The Netherlands; 6 Donders Institute for Brain, Cognition and Behaviour, Radboud University Nijmegen, Nijmegen, The Netherlands; 7 Institute of Human Genetics, University Hospital of Cologne, Cologne, Germany; 8 Dept Ophthalmology, Radboud University Nijmegen Medical Centre, Nijmegen, The Netherlands; 9 Servicio de Oftalmología, Hospital Universitario La Fe, Valencia, Spain; 10 Departamento de Oftalmología, Facultad de Medicina, Universidad de Valencia, Valencia, Spain

## Abstract

**Purpose:**

It has been demonstrated that mutations in deafness, autosomal recessive 31 (*DFNB31)*, the gene encoding whirlin, is responsible for nonsyndromic hearing loss (NSHL; DFNB31) and Usher syndrome type II (USH2D). We screened *DFNB31* in a large cohort of patients with different clinical subtypes of Usher syndrome (USH) to determine the prevalence of *DFNB31* mutations among USH patients.

**Methods:**

*DFNB31* was screened in 149 USH2, 29 USH1, six atypical USH, and 11 unclassified USH patients from diverse ethnic backgrounds. Mutation detection was performed by direct sequencing of all coding exons.

**Results:**

We identified 38 different variants among 195 patients. Most variants were clearly polymorphic, but at least two out of the 15 nonsynonymous variants (p.R350W and p.R882S) are predicted to impair whirlin structure and function, suggesting eventual pathogenicity. No putatively pathogenic mutation was found in the second allele of patients with these mutations.

**Conclusions:**

*DFNB31* is not a major cause of USH.

## Introduction

Usher syndrome (OMIM 276900–2; OMIM 276905; OMIM 605472) is the most common cause of genetic deafblindness. This syndrome follows an autosomal recessive pattern of inheritance and is characterized by retinitis pigmentosa (RP), sensorineural hearing impairment, and in some cases vestibular dysfunction. Three clinical types can be distinguished [1]. Patients with Usher syndrome type I (USH1) show severe to profound congenital hearing loss, early onset RP, and vestibular areflexia. Patients with type II (USH2) suffer from moderate to severe congenital hearing loss, onset of RP around puberty or in adulthood, but normal vestibular function. Usher type III (USH3) patients present with progressive hearing loss, RP, and variable vestibular function. Furthermore, some Usher syndrome patients cannot be classified into any of these three subtypes and are considered atypical Usher (USHA) patients. To date, nine genes are known to be involved in USH: *MYO7A* (USH1B), *USH1C, CDH23* (USH1D), *PCDH15* (USH1F), and *USH1G* for USH1; *USH2A*, *GPR98* (USH2C), and *DFNB31* (USH2D) for USH2; and *USH3A* for USH3 [2-14]. The eight genes responsible for USH1 and USH2 encode proteins that interact in a functional network.

The *DFNB31* gene was initially found to be responsible for recessive nonsyndromic sensorineural hearing impairment (NSHI), underlying the *DFNB31* locus [15]. *DFNB31* comprises 12 exons and encodes the whirlin protein. Two isoforms of whirlin are known. The short isoform is encoded by exons 6–12 and contains one PDZ domain (PDZ3) and a proline-rich region. The long isoform is encoded by all 12 *DFNB31* exons and contains three PDZ domains (PDZ1, PDZ2, and PDZ3) and a proline-rich region [15]. PDZ1 and PDZ2 have been shown to interact with usherin, myosin VIIa, G-Protein coupled Receptor 98 (GPR98), Scaffold protein containing Ankyrin repeats and SAM domain (SANS), and myosin XVa, while PDZ3 interacts only with myosin XVa [16-18]. All these findings made *DFNB31* an excellent candidate for Usher syndrome. More recently, Ebermann et al. [14] described a novel genetic subtype for Usher syndrome type II (USH2D) caused by truncating mutations in the long isoform of whirlin.

The protein whirlin is thought to play an important role in the elongation of stereocilia and development of cochlear hair cells and has been demonstrated to be the major scaffold protein of an Usher protein network in the ankle link complex of inner hair cells [15,17,19]. In the retina, whirlin is a key partner of another USH interactome in the periciliary region of photoreceptor cells. This Usher protein network is hypothesized to be essential for the regulation of cargo transfer from the photoreceptor inner segment to the outer segment by the ciliary transport system [18].

The recent finding that mutations in *DFNB31* cause USH2 prompted us to screen the 12 exons of this gene in 195 patients from diverse origins who have USH1, USH2, and USHA. Our aim was to elucidate the prevalence of *DFNB31* mutations in the pathogenesis of USH.

## Methods

### Patients

We included 149 patients with USH2, 29 patients with USH1, and six patients with USHA in this study. Furthermore, 11 unclassified USH patients were included from whom clinical data were not available. Of the 195 unrelated patients, 74 were of Spanish origin, 47 of German, 39 of Dutch, 17 of Hungarian, and 13 of other different origins (see Table 1).

**Table 1:** Clinical classification of USH patients.

**Origin**	**USH1**	**USH2**	**USHA**	USH NC*	**Total**
The Netherlands	0	27	2	10	39
Spain	21	51	2	0	74
Germany	2	44	0	1	47
Hungary	2	13	2	0	17
Canada	1	4	0	0	5
Russia	1	2	0	0	3
Arabia	0	1	0	0	1
Egypt	2	2	0	0	4
Turkey	0	3	0	0	3
Unknown	0	2	0	0	2
Total	29	149	6	11	195

*USH NC: Clinical data not available to classify the patients into any subtype.

Most of these patients were selected for *DFNB31* screening after discarding the presence of pathologic mutations in the most prevalent USH genes. Of the 195 patients, 116 patients correspond to those briefly mentioned in previous studies [13,16]. Clinical diagnosis of Usher syndrome patients was performed in different hospitals according to standard ophthalmologic, otorhinolaryngologic, and electroretinographic procedures. Written informed consent was obtained for each participant, and the study was approved by the review board of the Ethics Committee of each institution that participated in this work.

### Mutation screening

Venous blood samples were obtained and genomic DNA was extracted by standard protocols [7]. For mutation analysis of *DFNB31*, the 12 coding exons were PCR amplified using primers located in flanking introns and untranslated region (UTR) sequences (Table 2). PCR products were directly sequenced using standard procedures (Table 2). The obtained sequences were compared with the consensus sequence NM_015404.

**Table 2:** Primer sequences for the amplification of the coding region of *DFNB31*.

**Primer name**	**Sequence (5′-3′)**	**Annealing temperature**
Exon 1	F: CAGCAGCCAACTCTTGTGTC	55 °C
	R: CCAGAAAGGCCAAGTGATTC	
Exon 2	F: ACTCCCCAAATCCAAGTTCC	58 °C
	R: CAGAACCAGCCTCTTCTTGC	
Exon 3	F: CTCCTTGCCAGTCGGATAAG	55 °C
	R: GAGTGCTGATTGCTCTGCTG	
Exon 4	F: ATAAGGGGACCCTTGGAATG	55 °C
	R: TCCCCACTTTTTGGATGAAG	
Exon 5	F: GTCCGGAGTTTCCTTTACCC	55 °C
	R: TGGTCTGCTCTGTTCATTGC	
Exon 6	F: TGGCAATGAACAGAGCAGAC	58 °C
	R: GGAGGGCTTGTGAAGATGAC	
Exon 7	F: GACAGGGAAGCAGGAGTGAG	58 °C
	R: GATTCGAACTCAGGCTGGTC	
Exon 8	F: CAGCATCTCTGGCAGTTCAG	58 °C
	R: GGCTGTCATGGAGAGGAGAG	
Exon 9	F: GTGACAAGCTCTGGCTGATG	58 °C
	R: TTCAAACTGGGGTCTCCAAC	
Exon 10	F: GGTCTGGTTGAAAGGACAGG	58 °C
	R: GGCCTCCAGATTCCTAATCC	
Exon 11	F: GAGGCTGAGATTGGTCTTGG	55 °C
	R: CCTAGGTCTGCCCTTGAGTG	
Exon 12	F: CCCTTTCTCAGCATCTCCAG	58 °C
	R: GTCTGCCTTGTCCTGCTCTC	

Amplification conditions were 95 °C 5 min followed by 35 cycles of 30 s at 95 °C, 30 s at an annealing temperature specific for each exon (column 3) and 30 s at 72 °C.

Isocoding and missense changes found in a low frequency were analyzed with the program RESCUE-ESE (Christopher Burge Laboratory, Massachusetts Institute of Technology; Cambridge, MA). This program predicts the creation or elimination of exonic splicing enhancer sites (ESEs).

To assess the effect of the amino acid substitutions on the protein and to study the degree of interspecies amino acid conservation we used the software Alamut v1.5 Interactive Biosoftware (Rouen, France).

Intronic, isocoding, and missense changes were also analyzed using the programs NNSPLICE (Berkeley Drosophila Genome Project [BDGP] University of California Berkeley; Berkeley, CA) and Splice View (Institute of Biomedical Technologies, Milano, Italy) to predict if those changes could be affecting, creating, or eliminating donor/acceptor splice sites.

## Results

Mutation screening of *DFNB31* in 195 USH patients of diverse origins identified 38 different sequence variants: eight synonymous, 15 missense, and 15 intronic variants. Most of these changes are clearly polymorphic as they were detected in high frequencies and represent known single nucleotide polymorphisms (SNPs). Twelve sequence variants were found only once in our series, five changes were found twice, one variant was identified in three alleles, and another one in four (see Table 3). None of these 19 rare changes were found in the homozygous state or in trans to another putatively pathogenic variation.

**Table 3:** Variants detected in this study

**Nucleotide change**	**SNP**	**Exon**	**Amino acid change**	**Protein location**	**Number of alleles**	**Allele frequency**	**Homozygotes**	**Heterozygotes**	**Origin**
**Isocoding**									
c.117G>A	rs2297815	1	None	LC1-PDZ1	111	0.383	20	71	N, S, G, H, C, R, T, A, E
c.1353T>Ca	rs4979387	6	None	PDZ2-LC2	263	0.674	96	71	N,S, G, H, C, R, T, E
c.1455G>A		7	None	PDZ2-LC2	4	0.01	0	4	N, G
c.1486C>T		7	None	PDZ2-LC2	1	0.003	0	1	Unknown
c.1515G>A	rs34252199	7	None	PDZ2-LC2	10	0.026	0	10	S, G, H
c.1886G>A		9	None	LC3	18	0.046	6	6	S, N
c.2283C>T	rs34963246	10	None	LC3-PDZ3	30	0.077	3	24	S, G, H, S, C, T
c.2307C>T		10	None	LC3-PDZ3	2	0.005	0	2	S, G
**Missense**									
c.229A>T	rs56204273	1	p.T77S	Between LC1-PDZ1	1	0.003	0	1	N
c.1048C>T		4	p.R350W	PDZ2	1	0.003	0	1	E
c.1148C>A		4	p.T383N	Between PDZ2-LC2	2	0.005	0	2	N
c.1148C>G		4	p.T383S	Between PDZ2-LC2	1	0.003	0	1	S
c.1309G>A		6	p.A437T	Between PDZ2-LC2	6	0.015	0	6	S
c.1318G>A	rs4978584	6	p.A440T	Between PDZ2-LC2	88	0.226	16	55	N, S, G, C, R, T, E
c.1339G>A	6	p.D447H	Between PDZ2-LC2	1	0.003	0	1	H	
c.1684C>G	rs12339210	8	p.P562A	Between LC2-LC3	39	0.1	2	35	N, S, G, H, C, R, T, E
c.1838T>Ca	rs942519	9	p.M613T	Between LC2-LC3	179	0.459	45	89	N, S, G, H, C, R, T, E
c.1884C>A	9	p.S628R	LC2	1	0.003	0	1	E	
c.1943C>A	9	p.S648Y	Between LC3-PDZ3	1	0.003	0	1	S	
c.2169G>A	9	p.M723I	Between LC3-PDZ3	1	0.003	0	1	H	
c.2348T>C*	rs2274159	10	p.V783A	Between LC3-PDZ3	153	0.392	30	93	N, S, G, H, C, R, T, E
c.2388C>A	rs2274158	10	p.N796K	Between LC3-PDZ3	85	0.218	11	63	N, S, G, H, C, R, T, E
c.2644C>A		12	p.R882S	PDZ3	1	0.003	0	1	S
**Intronic**									
c.619–41A>G		2	None		1	0.003	0	1	H
c.837+41A>G		2	None		2	0.005	0	2	E, unknown
c.964–21A>G	rs2274163	4	None		114	0.292	14	86	N, S, G, H, C, C, R, T, E
c.1203+114C>T		5	None		2	0.005	0	2	S
c.1416+151A>G	rs4979385	6	None		19	0.049	2	15	S
c.1416+62delC		6	None		1	0.003	0	1	S
c.1416+22A>T	rs4979386	6	None		77	0.197	8	61	N, S, G, H, C, T, E
c.1627–12G>A	rs2274160	8	None		68	0.174	5	19	N, S, G, H, C, R, T, A, E
c.2236+84G>T	rs10982200	9	None		8	0.021	0	8	G, H
c.2237–44C>T	rs766835	10	None		3	0.008	0	3	N
c.2418+142A>G	rs10739410	10	None		29	0.074	5	19	N, S
c.2419–199A>G		11	None		18	0.046	5	8	N, S
c.2419–118G>A	rs55833018	11	None		12	0.031	0	12	S
c.2419–16T>C		11	None		1	0.003	0	1	G
c.2644–157C>T		12	None		2	0.005	0	2	G, S

The asterisk indicates previously reported as polymorphism by Tlili et al. [20]. Abbreviations: N represents The Netherlands, S represents Spain, G represents Germany, H represents Hungary, C represents Canada, R represents Russia, T represents Turkey, A represents Arabia), E represents Egypt, LC represents Low complexity protein domain.

### Isocoding changes

Only three out of the eight silent changes were found with an allele frequency ≤0.01 (see Table 3 for more details). Variant c.1486C>T was only found in one USH2 patient from uncertain origin, and c.2307C>T was found in one USH1 patient from Spain and in one USH2 patient from Germany. Variant c.1455G>A was detected in four alleles of patients from the Netherlands and Germany. These three rare isocoding variants were analyzed using the RESCUE-ESE program, but none were found to create or eliminate any ESEs.

These three changes were also analyzed with NNSPLICE and Splice View. Both programs predicted the creation of one new donor site when introducing the change c.2307C>T (NNSPLICE score of 0.66 [0.00–1.00] and Splice View score of 86 [0–100])

### Missense changes

From the 15 missense variants detected, only p.T77S, p.R350W, p.T383N, p.T383S, p.D447H, p.S628R, p.S648Y, p.M723I, and p.R882S showed a frequency ≤0.01 (see Table 3).

These nine variants were analyzed using the Alamut program. For two of these changes (p.R350W and p.R882S), this program predicted possible implications in protein structure and function. The amino acid substitution p.R350W (c.1048C>T) affected a highly conserved nucleotide at the cDNA level with a score of 0.9 (0–1). The amino acid conservation among 13 species was moderate, and the Grantham distance between amino acids Arg and Trp was 101 (0–215). Furthermore, this variation was found to be located in the PDZ2 protein domain. The change p.R882S (c.2644C>A at the cDNA level) affects a highly conserved nucleotide (score 1.0 [0–1]) and an amino acid highly conserved up to the fruit fly (considering 13 species). The physicochemical difference between Arg and Ser was found to be moderate since a Grantham distance of 110 (0–215) was obtained. Also this variation was located in the PDZ3 domain.

These nine rare missense variants were further evaluated for an effect on exonic splice enhancers by using the RESCUE-ESE program. The change c.1148C>A (p.T383N), found in two unrelated USH2 patients from the Netherlands, creates four new putative ESE sites, whereas c.1339G>A (p.D447H), detected in one USH2 patient from Hungary, suppresses one existing ESE sequence and creates two new putative ESE sites. The remaining variants were not found to affect any ESE site.

These changes were also analyzed with NNSPLICE and Splice View, but neither was found to affect, create, or eliminate any donor or acceptor splice site.

### Intronic changes

All intronic variants were detected in frequencies ≥0.01, except for c.619–41A>G, c.837+41A>G, c.1203+114C>T, c.1416+62delC, c.2237–44C>T (rs766835), c.2419–16T>C, and c.2644–157C>T (Table 3). None of these changes were predicted to affect or create splice donor or splice acceptor sites in the analysis with the NNSPLICE and Splice View programs.

## Discussion

To date only one Usher family with clearly pathogenic, truncating, *DFNB31* mutations has been described [14]. Here, we describe additional sequence variants in USH patients tested for mutations in *DFNB31* and discuss their putative pathologic effects.

A total of 38 different variants were found in the *DFNB31* sequence, but no variant was a frameshift or a nonsense change. Out of these, 19 rare variants were found with allelic frequency ≤0.01; two of them were located in PDZ protein domains (p.R350W and p.R882S), and four were predicted to create or abrogate splice sites (c.2307C>T, p.T383N, p.D447H, and p.R882S).

By the introduction of p.R350W and p.R882S, uncharged amino acids may affect the three-dimensional structure of the protein, which is important for the adequate function of interacting domains. Furthermore, both the amino acids R350 and R882 were well conserved throughout evolution, indicating these residues have an important role in protein structure and function. All these factors point to a possible pathologic effect for these two variants.

Segregation analysis for the variants p.R350W, p.G769G, p.T383N, and p.D447H could not be performed. In some cases, we only had a DNA sample from a patient without any family history. In other cases, the patient was a sporadic case and segregation analysis did not reveal any information about the pathogenicity of the variant (e.g., in the case of p.R882S). We found variant p.R882S in a sporadic Spanish USH2 patient together with the variant p.S648Y. Segregation analysis for these changes was performed in healthy relatives and revealed that both variants were located on the same allele. In addition, p.R882S and p.S648Y were present in the patient, in his healthy brother and sister, and in his two healthy children.

To explore the possibility of yet unidentified exons of *DFNB31*, which may contain mutations in the second allele of patients with a potentially pathogenic mutation on one allele, we performed an in silico search by using Geneid (Genome Bioinformatics Research Lab; Center for Genomic Regulation, Barcelona, Spain), N-SCAN (Computational Genomics Laboratory; Washington University of St. Louis, St. Louis; MO) and we also searched in the UCSC Genome Browser (University of California Santa Cruz Genome Bioinformatics Web Site, Santa Cruz, CA), but no additional exons were predicted in the DFNB31 locus.

In summary, this study did not reveal evidence for the involvement of *DFNB31* mutations in 195 unrelated USH patients. However, total or partial gene deletions and duplications can escape the screening method applied herein and therefore cannot be excluded. Our results indicate a minor causative role for *DFNB31* in Usher syndrome. However, modifying effects of the variants we detected might contribute to the phenotype of Usher syndrome.

Regarding the implication of this gene in nonsyndromic hearing loss, the studies performed so far also indicate that DFNB31 is a rare form of deafness [15,20]. We previously hypothesized that *DFNB31* mutations may also be causative for nonsyndromic retinal degenerations. This remains to be confirmed.
